# Fungiculture or Termite Husbandry? The Ruminant Hypothesi

**DOI:** 10.3390/insects3010307

**Published:** 2012-03-16

**Authors:** Tânia Nobre, Duur K. Aanen

**Affiliations:** Laboratory of Genetics, Wageningen University, Droevendaalsesteeg 1, Radix West, Building 107, 6708 PB Wageningen, The Netherlands; E-Mail: duur.aanen@wur.nl

**Keywords:** fungus-growing termites, *Termitomyces*, Macrotermitinae, gut microbiota, lignocellulose, host-symbiont specificity, ruminant hypothesis

## Abstract

We present a new perspective for the role of *Termitomyces* fungi in the mutualism with fungus-growing termites. According to the predominant view, this mutualism is as an example of agriculture with termites as farmers of a domesticated fungus crop, which is used for degradation of plant-material and production of fungal biomass. However, a detailed study of the literature indicates that the termites might as well be envisioned as domesticates of the fungus. According to the “ruminant hypothesis” proposed here, termite workers, by consuming asexual fruiting bodies not only harvest asexual spores, but also lignocellulolytic enzymes, which they mix with foraged plant material and enzymes of termite and possibly bacterial origin. This mixture is the building material of the fungus garden and facilitates efficient degradation of plant material. The fungus garden thus functions as an external rumen for termites and primarily the fungi themselves benefit from their own, and gut-derived, lignocellulolytic enzymes, using the termites to efficiently mix these with their growth substrate. Only secondarily the termites benefit, when they consume the degraded, nitrogen-enriched plant-fungus mixture a second time. We propose that the details of substrate use, and the degree of complementarity and redundancy among enzymes in food processing, determine selection of horizontally transmitted fungal symbionts at the start of a colony: by testing spores on a specific, mechanically and enzymatically pre-treated growth substrate, the termite host has the opportunity to select specific fungal symbionts. Potentially, the gut-microbiota thus influence host-fungus specificity, and the selection of specific fungal strains at the start of a new colony. We argue that we need to expand the current bipartite insect-biased view of the mutualism of fungus-growing termites and include the possible role of bacteria and the benefit for the fungi to fully understand the division of labor among partners in substrate degradation.

## 1. Introduction

Mutualistic symbiosis has played a major role in evolution. Through synergy of combined abilities, a mutualistic alliance between two or more species can adapt faster than individual organisms [1]. For example, a symbiosis between a proto-eukaryotic cell and endosymbiotic prokaryotes is at the basis of eukaryotes, which eventually led to protists, plants, fungi and animals [2]. Another example is the symbiosis between plants and arbuscular mycorrhiza, which allowed the colonization of land by plants, fundamentally altering environmental conditions on earth [3].

Insects represent the most diverse animal group in the terrestrial ecosystem. Over one million species of insects have been described, but current estimates of total insect diversity vary from 3–80 million species [4,5,6]. Although most adaptive radiations of insects are likely to have been driven by flight (*i.e.*, efficient dispersal), resistance to desiccation, voltinism and metabolic adaptations to herbivory, their capacity to engage in symbiosis may have contributed to their enormous evolutionary success [5]. It is estimated that 20% of all insects are obligatorily associated with symbiotic microorganisms, such as endosymbiotic bacteria and gut bacteria, allowing these groups to specialize on imbalanced food sources [7,8]. The best characterized associations are with bacteria that provide nutritional benefits to their host, such as the association between aphids and *Buchnera* bacteria [9,10]. *Wolbachia spp.* are best known for their manipulation of host reproduction [11], but recent work has shown that they sometimes also offer protection against parasitism and pathogens [12]. In particular, insects that feed on incomplete specialized diets, such as plant sap, vertebrate blood or woody materials, tend to engage in symbioses with partners that provide essential nutrients and/or help them in food digestion. 

In insect mutualisms with fungi, the insects generally consume their symbionts as a food source, and as a source of enzymes for degradation of plant material. Behaviorally complex systems of such insect agriculture have only evolved in three insect orders: once in termites [13], once in ants [14] and seven times in ambrosia beetles [15]. In this paper, we discuss recent work on fungus-growing termites. We focus on the unresolved debate about the exact role in substrate degradation of the symbiotic fungus. We propose a new hypothesis for the division of labor between termites and fungi in (ligno)cellulose degradation. We also discuss the potential role of bacteria, which have been studied in much less detail, and their potential to mediate host-fungus specificity.

## 2. The Mutualism between Macrotermitine Termites and *Termitomyces* Fungi

Termites of the subfamily Macrotermitinae (Termitidae) live in an obligate mutualistic symbiosis with fungi of the genus *Termitomyces* (basidiomycetes). Fungus-growing termites are restricted to the Old-World tropics, with the highest diversity in African tropical rain forests. This habitat has also been reconstructed as the centre of origin [16], likely just before the expansion of the savannah, about 31 Ma [17]. Entering the symbiosis has allowed the fungi to overcome highly unfavorable seasonal conditions (e.g., temperature fluctuations and low moisture), and the termites to exploit complex plant substrates (e.g., lignocellulose). The current habitat of fungus-growing termites ranges from rain forest to savannah. Whereas species numbers of fungus-growing termites are highest in rainforest habitats [17,18], their relative contribution to ecosystem decomposition is highest in savannahs, with up to 20% of all carbon mineralization attributed to fungus-growing termites [19]. The highest estimate on their contribution to carbon mineralization, 90%, relates to their activity in a very arid habitat [20]. The transition to fungiculture in both partners has occurred only once, with no reversals to free-living states of any of the two partners [13].

**Figure 1:**
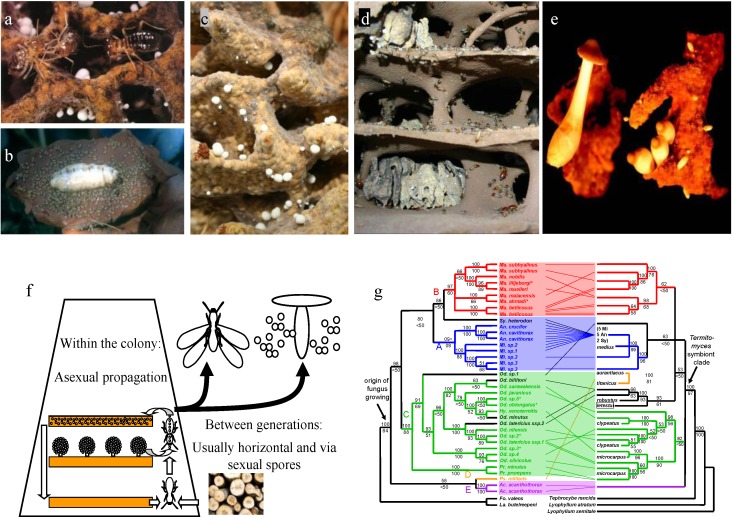
Aspects of the mutualism between fungus-growing termites and *Termitomyces *fungi, using *Macrotermes* as a model system. (**a**) Close-up of a fungus garden with multiple asexual fruiting bodies (nodules). The spores survive passage through the termite gut and are mixed with predigested plant material to be deposited as inoculated fresh garden substrate [17,18]; (**b**) the queen and king in an opened royal chamber; (**c**) A fungus garden showing freshly-deposited substrate on top (dark), multiple nodules in mature parts (white structures), and degraded lower parts; (**d**)the inside of a colony showing the interconnected network of chambers that contain fungus gardens, containing a mass of masticated plant substrate in which *Termitomyces* grows (the fungus comb); (**e**) Mushrooms of various developmental stages of a colony *Macrotermes natalensis*; (**f**) Key aspects of the life cycle of a typical species of fungus-growing termites with horizontal transmission [23]. The fungus is clonally propagated via asexual spores inside the colony [24], which can live for more than 20 years. Dispersal occurs via the yearly production of sexual offspring, independently in termites (alates) and fungi (sexual spores from mushrooms); (**g**) Patterns of coevolution between fungus-growing termites (left) and their associated *Termitomyces* fungi (right) [10].

Fungus-growing termites are eusocial insects, with division of labor among colony members in brood care and reproduction, and overlap of adult generations [21]. Colonies of fungus-growing termites are normally founded by a single reproductive pair (Figure 1b). When the workers of the first brood are mature they leave to forage and usually acquire a specific fungus from the environment [22]. 

Presumably, along with their foraged plant material, they will collect basidiospores, which will be the inoculum of the new fungus garden. The majority of species have this horizontal transmission, implying that reproduction and dispersal of the two partners occurs independently [25]. However, two independent transitions have occurred towards vertical symbiont transmission (*i.e.*, where *Termitomyces *vegetative spores are transported in the gut of alates from a parental colony and used to inoculate the fungus comb of the newly founded colonies). In both cases, vertical transmission is uniparental, but via different sexes: via the female alate in all studied species of the genus *Microtermes* and via the male alate in the species *Macrotermes bellicosus* [13,26,27,28,29].

The symbiotic fungus grows as a single-strain monoculture [24,30] on a special substrate, the fungus comb, maintained by the termites through continuous addition of pre-digested plant material (Figure 1a,c,d). The consumption of comb material varies between species. In some species, there is a continuous turnover of comb material, with primary feces (including fungal inoculum) being deposited on top and the older comb material at the bottom being consumed (e.g., species within *Macrotermes* and *Odontotermes*). In other species, for example in species of the genus *Pseudacanthotermes*, the entire comb is consumed before building a new one in an empty chamber [31].

Fungus-growing termites show moderate specificity with their symbionts: monophyletic groups of the termites (*i.e.*, usually either a single genus or several genera) are associated with monophyletic groups of fungi [13,32]. However, there are also signatures of host switching among certain divergent species over evolutionary time, so that termite species can be associated with different clades of fungal symbionts [17,33,34,35]. This is consistent with horizontal symbiont transmission as the ancestral and still most common transmission mode in this symbiosis (Figure 1f).

## 3. Large Differences in Host-Fungus Specificity

Although strict vertical transmission should lead to a high degree of host specificity, the fungal symbionts of species of fungus-growing termites with this transmission mode (Figure 1g) are not monophyletic [13,29,32,35]. Therefore, occasional events of horizontal transmission must be inferred for these species. Calibrated phylogenetic reconstructions show that species of the genus *Microtermes* are associated with symbionts of a relatively recent origin (about twice as young as the host genus) and one of the symbionts associated with *Microtermes *is highly successful and has dispersed across multiple divergent genera [17]. *Macrotermes bellicosus* is associated with at least four symbiont lineages, which do not constitute a clade [13,29]. Furthermore, we have recently demonstrated that the pool of fungal symbionts reared and transmitted by *M. bellicosus* is not genetically isolated from the *Termitomyces* pool of other *Macrotermes* species and that occasional recombination occurs among the fungal symbionts of *M. bellicosus*. Thus, although the population structure of *M. bellicosus *fungi has a clear signature of clonal propagation, occasionally recombination and exchange with fungi associated with other *Macrotermes* species occurs [29]. In contrast, high specificity is sometimes found in lineages with horizontal transmission. For example, all colonies of the fungus-growing termite *Macrotermes natalensis* studied so far are associated with a single, sexually reproducing, horizontally transmitted lineage of *Termitomyces *symbionts [33,36].

Transmission mode, therefore, does not explain observed host-fungus specificity patterns very well. Therefore, interaction specificity must depend on other factors than transmission mode. One such factor may be the role of the fungal symbiont.

## 4. Hypotheses on the Role of the Symbiotic Fungus

**Figure 2:**
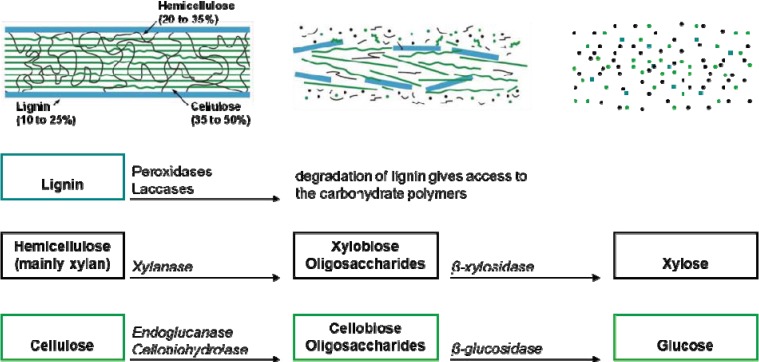
Schematic overview of the composition of secondary plant-cell walls of woody tissue and grasses, which are composed predominantly of lignin, hemicellulose and cellulose. Some of the most important enzymes involved in the degradation of the main polysaccharides into disaccharides and oligosaccharides, which are further degraded to soluble monosaccharides that can be assimilated, are shown.

Fungus-growing termites mainly feed on dead plant material, that is to say they mostly ingest lignocellulose, the major component of plant cell walls (Figure 2). The ratios between various constituents of plant cell walls vary between plant species, but even within a single species they may also vary with age, stage of growth, and other conditions. However, cellulose is usually the dominant structural polysaccharide of plant cell walls (35–50%), followed by hemicellulose (20–35%) and lignin (10–25%) [37] with the cellulose and hemicellulose polymers tightly bound to the lignin [38]. These substances are recalcitrant, and many different enzymes are involved in their degradation.

The high efficiency of fungus-growing termites in digesting these plant substances has been attributed to their symbiosis with fungi. However, the exact role of the fungal symbiont is still debated. Overall, the proposed functions fall into the following categories, which are not mutually exclusive [22,39,40,41,42]:

(1) *Termitomyces* has a role in lignin degradation, which enhances the digestibility of the cellulose (proposed by Grassé and Noirot [43]);
(2) *Termitomyces* is an additional protein-rich food source (mainly the fungal nodules);
(3) *Termitomyces* provides cellulases and xylanases to work synergistically and/or complementarily with endogenous termites enzymes [44,45].

Although understanding the role of the symbiotic fungus, and variation therein, is crucial to understand the evolution and stability of this mutualism, only few studies have addressed this question. Most studies have focused on the role of the fungus in cellulose digestion for the termites, and are mainly based on species of the genus *Macrotermes*. Moreover, these studies are difficult to compare because they had different foci, were based on different species and/or used critically different methodologies. A complicating factor is that the role of the fungus differs between genera and species [31,45,46,47], and may even vary among symbiont strains within species (TN, unpublished data).

Physical disruption of the lignocellulose by insect mastication and chemical disruption of the lignin aids the access of cellulases to the cellulose substrate. Hyodo and co-workers [47] found evidence for the lignin hypothesis, originally proposed by Grassé and Noirot [35], based on chemical composition of fungus combs of *Macrotermes annandalei*, *M. gilvus* [48], *M. carbonarius* and *M. muelleri*. Rohrmann [49], also showed a decreasing lignin content in old fungus comb of *Macrotermes ukuzii* and *M. natalensis*, consistent with a role in lignin degradation for the fungal symbionts of these species.

However, lignin degradation does not seem to be the main role of the symbiotic fungi in all species of fungus-growing termites. For example, lignin degradation was shown to be rather inefficient [47] in *Odontotermes* -*O*. sp.1, *O. formosanus*, *O. takensis* and *H. makhamensis* (belonging to the Asiatic genus *Hypotermes* derived from *Odontotermes*; [8])- *Ancistrotermes pakistanicus* and *Pseudacanthotermes militaris*.

Next to the capabilities to degrade lignin attributed to *Termitomyces* associated with *Macrotermes* [47], cellulolytic and xylanolytic activities have also been ascribed to *Termitomyces*. *Termitomyces* nodules associated with *Macrotermes subhyalinus* contain specific cellulase activity [50]. Likewise, *Termitomyces *strains associated with *M. bellicosus* and *M. mulleri* were shown to have cellulolytic activity [31,45,51]. However, Veivers and co-workers [41] found that the amount of enzyme produced by the symbiotic fungi associated with *M. michaelseni* and *M. subhyalinus* was not sufficient to account for the measured respiration of the workers. Some studies have shown xylanolytic activity of *Termitomyces* associated with *M. bellicosus* [45,52] and endo- and exoxylanases have been isolated from the fungal symbiont of this host [53]. Also from the *Termitomyces* sp. associated with *M. subhyalinus* endoxylanases have been isolated and purified [54].

In contrast, in *Odontotermes formosanus*, cellulase activity has been found to be significantly higher in workers than in the symbiotic fungus [55] and the termites themselves seem to be able to excrete cellulase [56]. From the synergy of the different cellulases, a higher activity efficiency in degrading the substrate could emerge, although the low cellulase activity found in the fungus suggests that the main role of the cultivated fungus for this species is not in cellulase degradation. The fungus *Termitomyces clypeatus *(hosted by *O. lacustris *[42] but likely also with other *Odontotermes* species—see phylogenetic reconstruction in [13]), has been shown to be able to produce a broad spectrum of extracellular glucosidases (cellulase, sucrase, cellobiase *etc*., [57]), but also of xylanases [58,59] and laccases [60].

**Figure 3:**
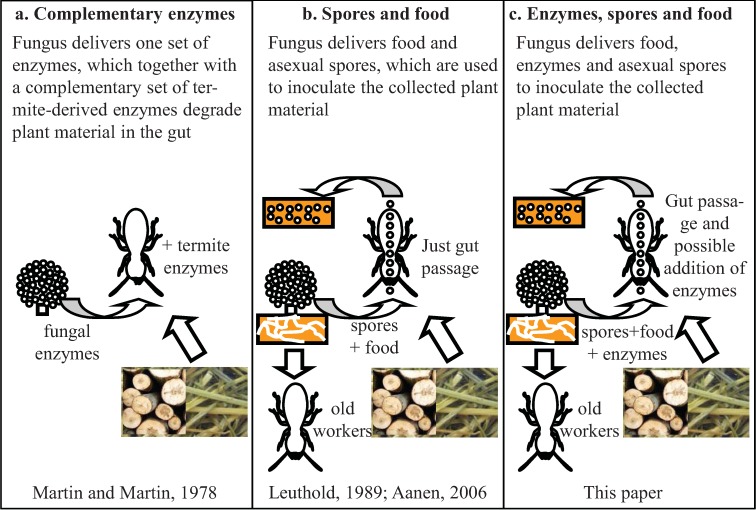
Alternative hypotheses for the role of the fungus as a source of food, spores and enzymes. (**a**) According to the acquired enzyme hypothesis, fungi and termites deliver different sets of cellulolytic enzymes, which together degrade the plant material inside the gut; (**b**) Nodules are an additional protein-rich food source and are used to inoculate the fungus garden; (**c**) the ruminant hypothesis—here raised—where nodules are a source of food, enzymes and spores. The fungi may deliver the complete set of (ligno)cellulolytic enzymes, or the fungi and termites deliver different, complementary sets.

Martin and Martin, studying substrate degradation in *M. natalensis*, proposed the acquired enzymes hypothesis (Figure 3a) [44]. According to this hypothesis, *Termitomyces* provides cellulases and xylanases, which complement (or work synergistically with) the endogenous enzymes in the termite gut. Consistent with this hypothesis, high concentrations of fungal enzymes were found in other species of *Macrotermes *[45,50,51]. However, in *M. michaelseni* and *M.bayerni*, enzymes of fungal origin were scarcely available in the guts of termite workers suggesting that most cellulolytic enzyme activity was of termite origin [41,61,62]. Furthermore, a division of labor between termite workers has been demonstrated in some species [63,64], with younger workers consuming nodules and tending the comb structure and older workers feeding on degraded fungus comb and foraging for plant material. Therefore, termite workers only have access to the fungal enzymes for a limited period of their life.

This criticism also applies to the hypothesis that *Termitomyces* is an additional protein-rich food source, mainly in the form of nodules. However, it could be imagined that growing younger workers need more protein-rich diets (Figure 3b). For *Ancistrotermes pakistanicus* and *Pseudacanthotermes militaris* [47], and for *Ancistotermes cavitorax *[45], where no lignin degradation capabilities have been demonstrated, the main role of the fungus was suggested to be a protein-rich food source. Next to a protein-rich food source, it is now well established that nodules also deliver spores, which can survive gut passage, for the inoculation of new comb fragments [24].

## 5. The Ruminant Hypothesis: Termites Helping the Fungus to Degrade Lignocellulose

Surprisingly, none of the current hypotheses explicitly addresses the question of fungal growth itself, and the growth substrate for the fungus. Instead, the fungus is often seen as an enzyme factory, used by the termites to accomplish cellulose degradation inside the termite gut. This view is probably influenced by the situation in other groups of termites, where the degradation of cellulose is achieved by gut bacteria and protozoa.

Here, we explicitly consider the advantage for both partners in the mutualism and propose a new hypothesis, the ruminant hypothesis. As first described by Swift [65], in an external rumen animals assimilate or digest the metabolic products of microflora associated with decomposing plant material. According to this view, the fungus garden would function as an external rumen for termites. Collins [66] already in 1983 described the symbiosis with the fungus as a way for termites to have an “external digestive system”, which decomposes their feces, decreasing the C/N ratio of foraged products by metabolizing carbohydrates prior to consumption.

It is now well-established that nodules deliver asexual spores used to inoculate fresh garden substrate [18,58]. Furthermore, it has been demonstrated for several species that the nodules contain enhanced concentrations of lingo-cellulolytic enzymes [44,67,68]. We propose that the first gut passage serves to mix fungal enzymes and spores with the substrate and with enzymes of termite and possibly bacterial origin (Figure 3c). This mixture facilitates efficient degradation of plant material. This means that the fungus uses the termites to efficiently spread ligno-cellulolytic enzymes, both fungus and insect derived (and putatively bacterial; see below).

An important prediction of this model is that enzymes are not secreted in large amounts by the mycelium, but accumulate in the nodules and become available upon partial digestion of cells during gut passage. Obviously, the ruminant hypothesis has a clear parallel with the acquired enzyme hypothesis [44,67]. However, a crucial difference between the two is the moment the termites benefit. According to the acquired enzyme hypothesis, there is a direct benefit of fungal-derived enzymes, which accomplish intestinal cellulose breakdown, thus making nutrients available for the termites. In contrast, according to the ruminant hypothesis, initially the fungi benefit from their own enzymes and from termite acquired enzymes using the termites to efficiently mix these enzymes with the substrate and their own asexual spores. The termites only secondarily profit from these enzymes in the form of degraded, nitrogen-enriched plant material and fungal biomass, when old fragments of the comb are consumed. The two main lines of criticism against the acquired enzyme hypothesis, viz. that the amounts of enzyme produced by the fungus are not sufficient, and that fungal growth itself is not explicitly considered, thus do not apply to the ruminant hypothesis.

Since the nodule, which presumably descends from one or a few cells, produces both spores and enzymes, this implies a strong link between phenotype (enzyme production) and genotype. This genotype-phenotype coupling should additionally provide a better protection against cheaters not producing enzyme, compared to excretion of enzymes by the mycelium as a common good, of which other genotypes can also profit.

## 6. Other Putative Nutritional Symbionts

It is well established that the *Termitomyces* symbiont contributes, in one way or another, to plant material decomposition, but as the host termites also produce their own cellulases, the exact division of labor between the two partners remains to be established. It has been assumed that the strongly aerobic fungal symbiont removes the need for microbial activity (particularly for their hydrolysing capabilities) in plant-material degradation, leading to the current bipartite view of the nutritional biology of the fungus-growing termites [69]. 

However, bacteria producing cellulolytic enzymes have been isolated from *Odontotermes* spp. as early as in the 50’s [70]. Reports on the isolation of cellulose- and xylose-decomposing bacteria from hind guts of fungus-growing termites, mainly from *Odontotermes* and *Macrotermes*, have been published since [71,72,73]. Earlier work on the gut-bacteria composition of Macrotermitinae have provided the first evidence that the microbiotas resemble those of other termites and that fermentation products are accumulated [72,74,75]. More recently, the diversity of gut bacteria in fungus-growing termites has been studied, using culture-independent techniques. Bacterial clones (from the Cytophaga-Flexibacter-Bacteriodes phylum) known to be specialized in the degradation of plant fibres and proteins [76], likely constituents of the termite diet, were reported to constitute the major part of the gut flora of *O.formosanus *[77], *M. gilvus* [78] and *M. michaelseni* [79] workers. Recently, lignocellulase-positive bacterial clones from *M. annandalei *have been isolated [80], directly demonstrating that the gut bacteria of fungus-growing termites contribute with glycosidases to digestion. However, the relative contribution of gut bacteria in fungus-growing termites to degradation of plant material remains to be established.

Whenever a comparison has been made between microbial diversity in the termite gut and in the fungus comb, higher numbers and greater diversity of bacteria have been found in the termite gut (*Macrotermes gilvus *[78]; *Odontotermes yunnanensis* [81]). These findings, showing a consistent and unique termite gut flora, suggest co-evolution of the gut bacteria with the termite host. Although the bacterial community of the termite gut is commonly exchanged between colony members and likely transmitted to the next generation vertically via trophallaxis, promoting coevolutionary diversification along with the host, differences in diets (even within the same feeding group) inevitably influence the composition of the microbial community [78,82]. The few data available so far do not allow us to conclude if the termite gut microbiota is specific to certain taxa of termites, feeding groups or a combination of both. However, at least some specificity exists, as all studies to date show that a single termite species harbors several hundred species of gut bacteria unique to termites. The increasing interest in the gut-bacterial community of fungus-growing termites as a source of lignocellulase genes [80,83,84] will likely stimulate research towards a better understanding of the functional details of coevolution and the role of the gut bacteria therein.

## 7. Selection of Specific Fungal Strains When Starting a Colony

Whereas the termite gut-microbiota can be assumed to be mainly vertically inherited, the fungal symbiont is transmitted horizontally in most species (reviewed in [25]). We have discussed that transmission mode is not a good predictor of interaction specificity and argued that the specific role of the fungal symbiont may instead be an important factor to explain the observed differences in interaction specificity between species. But how is the right fungal symbiont selected and maintained, when a new colony starts without a fungal symbiont?

All studies analyzing the diversity of *Termitomyces* within colonies have shown that individual colonies of fungus-growing termites are associated with a single strain of *Termitomyces*, irrespective of symbiont transmission mode [13,30,33,34]. In all genera studied so far, the termite workers harvest the fungal nodules containing asexual spores [85,86,87]. These spores are mixed with foraged plant material in the gut of workers and deposited on the fungus comb, allowing rapid growth of a new mycelium and of new nodules, which are then consumed again. This within-colony propagation mode is universal for the entire clade of fungus-growing termites, although the details of division of labor between workers differ between genera [24,88]. During this continuous asexual propagation within a colony, the fungus is thus reared as a monoculture.

With horizontally transmitted symbionts, the first fungus garden will usually be established from sexual spores collected from the environment together with foraged plant material. Under these circumstances there will be ample opportunity that multiple spores are brought in to the colony. Experiments have shown that a monoculture emerges due to positive frequency dependent selection, genetic drift and selection for rapid asexual spore formation [24,30]. Subsequent colonization by other strains, which will initially be in a minority, will thus be prevented. This results in a life-time commitment between a termite society and a single fungal clone, so that the costs of acquiring a non-optimal symbiont at the start of a colony will be high.

These experiments thus clearly show how starting from a mixture of spores a single monoculture emerges. However, these experiments were performed with a mixture of symbionts, which all were isolated from a single species, and thus all were of a certain specific genotype. A different question is how, from a mixture of potentially good and potentially unsuited genotypes, a *specific *fungus is selected. The foraged material, including *Termitomyces* basidiospores, is ingested by the termite foragers, brought into the nest and deposited as feces to form the new fungus comb. In this first gut passage, it is known that the foraged plant substrate is mixed with the spores but, as argued earlier in this paper, there is also ample potential to further pre-treat this substrate by mechanical and enzymatic action through gut-derived enzymes, either from termite or bacterial origin. Because this masticated plant-material contains the fungal inoculum and is going to be the growth substrate of the fungal symbiont, the host has the opportunity to screen its symbionts on this substrate. According to this novel hypothesis, the pre-treatment of substrate thus determines the fungal symbiont and accounts for the observed partner specificity. The differences in the “selection” media offered to the symbiotic fungus may depend on differences in host-derived enzymes, but also in host diet and in the gut microbial community.

## 8. Conclusions

In this paper we have proposed the ruminant hypothesis. According to this novel hypothesis fungal enzymes and spores produced in nodules are mixed with the foraged plant material and enzymes of termite and bacterial origin. This mixture initially benefits the growth of the fungus, and facilitates efficient degradation of plant material, ultimately also to the benefit of the termites in the form of a nitrogen enriched food source. Furthermore, the mutualistic diversification observed in the fungus-growing termites suggests that the evolution and maintenance of specific host-fungal symbiont interactions is determined by the demands of the termites on their fungal symbiont to fulfill their nutritional requirements. The exact role of the termite gut bacteria remains to be studied in more detail. The isolation of ligno-cellulolytic bacteria indicates that this question deserves more attention. The extent to which bacterial metabolism is critical for the degradation of the plant material still remains an open question, as well as the extent to which the bacteria are mediating host-fungal association at the start of a new colony. We have argued that the termite host has the opportunity to select specific fungal symbionts by testing spores on the offered growth substrate, which consists of masticated plant material that has undergone a first gut passage and is thus potentially subject to bacterial and termite derived enzymes. We have argued that we need a more balanced view on the mutualism of fungus-growing termites, moving away from its currently bipartite and insect-focused perspective, to include the possible relevance of bacterial symbionts and to emphasize the perspective of the fungus, in order to fully understand coevolution, specificity patterns and the long-time stability of this fascinating mutualism.
